# Relationship between COMT Gene Polymorphism, Anxiety, and Pain Perception during Labour

**DOI:** 10.3390/jcm12196298

**Published:** 2023-09-29

**Authors:** Jadranka Šanjug, Krunoslav Kuna, Marina Šprem Goldštajn, Lidija Fumić Dunkić, Andreja Carek, Dubravka Negovetić Vranić

**Affiliations:** 1 Department of Gynaecology and Obstetrics, General Hospital Zabok and War Veterans’ Hospital, Bračak 8, 49210 Zabok, Croatia; 2 Department of Gynaecology and Obstetrics, Sestre Milosrdnice University Hospital Centre, 10000 Zagreb, Croatia; krunoslav.kuna@kbcsm.hr; 3 Department of Obstetrics and Gynecology, Clinical Hospital Center Zagreb, 10000 Zagreb, Croatia; 4 Division of Anesthesiology, Intensive Care and Pain Medicine, Sestre Milosrdnice University Hospital Center, 10000 Zagreb, Croatia; lidija.fumic.dunkic@gmail.com; 5 Department of Fixed Prosthodontics, School of Dental Medicine, University of Zagreb, Gunduliceva 5, 10000 Zagreb, Croatia; 6 Department of Pediatric Dentistry, School of Dental Medicine, University of Zagreb, Gunduliceva 5, 10000 Zagreb, Croatia

**Keywords:** COMT gene polymorphism, labour, pain, anxiety, nitrous oxide

## Abstract

Background: The COMT gene polymorphism is associated with neurological and psychiatric disorders and pain perception. The present study investigates the existence of anxiety and pain perception in relation to the COMT (catechol-O-methyltransferase) gene polymorphism in labouring women (during “natural” childbirth) with or without inhaled analgesia. Methods: A total of 181 women who chose vaginal birth were enrolled in this study. To present the difference in pain perception, the parturients were divided into one group (*n* = 90) that chose labour analgesia with inhaled nitrous oxide (50% nitrous oxide and 50% oxygen) and one group (*n* = 91) without analgesia. The blood samples were taken during the pregnancy as a part of routine pregnancy controls in the hospital. The COMT gene polymorphism was detected with the PCR technique. The pain perception of parturients was self-evaluated two times according to the VAS (Visual Analogue Scale), and anxiety as a personality trait was determined with the STAI-T (State Trait Anxiety Inventory). Pain perception as well as anxiety were compared according to COMT genotypes. Results: In the 181 pregnant women, there were 40 women (22%) of wild homozygotes (GG) of COMT, 95 women (53%) of mutant heterozygotes (GA), and 46 women (25%) of mutant homozygotes (AA). A negative association of pain perception with the GA (mutant heterozygote) polymorphism of the COMT gene versus the wild-type (GG polymorphism) was observed. The GA polymorphism of the COMT gene was associated with 0.46 units lower pain perception compared to the wild type (GG). The anxiety trait score in group AA was lower than in groups GA and GG. The difference reached statistical significance only when comparing AA versus GA (*p* > 0.042). Analgesic efficacy of nitrous oxide was noticed in 22% of labouring women who reported moderate pain (VAS score 4–7). Conclusions: The COMT gene polymorphism was associated with pain perception and anxiety among parturients. The COMT gene polymorphism GA was associated with negative pain perception among labouring women. Nitrous oxide showed statistical significance in anxiolytic efficacy during labour in women with mild anxiety as a personality trait. Anxiolytic efficacy of nitrous oxide has shown better efficacy in parturients with the COMT gene polymorphism AA.

## 1. Introduction

Labour is considered to be one of the most painful experiences in women’s lives, and 35 to 58 percent describe it as severe or intolerable [1,2]. Perceived pain is the result of a number of different interactions involving psychological and physiological mechanisms. Women cannot predict the severity of pain, which can cause fear and anxiety. Pain in childbirth is primarily a subjective feeling, and its threshold varies from woman to woman. The evaluation of pain perception is based on the subjective perception of the parturient, previous pain experiences, their psychological condition, and their expectations regarding the pain and duration of childbirth. Despite that, many women still choose “natural childbirth” and try to delay or avoid epidural analgesia in childbirth.

Anxiety, fear, and pain during childbirth are very common occurrences. They can affect the change in hormone secretion, i.e., adrenocorticotropic hormone, cortisol, catecholamines, and endorphin, and a reduction in the pain threshold in labouring women. The deterioration of pain affects emotions [3]. The above phenomena result in a fear-tension-pain syndrome and form a vicious circle for pregnant women. Eventually, these adverse factors lead to complications during childbirth, such as increased dystocia and caesarean section rates [4,5]. Anxiety, as a personality trait measured by the STAI-T questionnaire, was used to assess how anxiety as a personality trait affects pain perception during labour and birth in parturients with analgesia and those who did not receive analgesia [6].

Inhaled nitrous oxide offers effective pain relief during labour for many women who opt for “natural birth”. The choice of analgesia should be safe for the mother and foetus [7,8]. Nitrous oxide has low blood solubility and anaesthetic potency; however, it helps women in labour cope with anxiety. The low blood solubility of inhaled nitrous oxide allows rapid onset and offset of action with peak brain concentrations at 60 s after inhalation without the risk of overdose [9]. With proper patient instruction, the nitrous oxide can be self-11010 administered with a peak effect at the peak of uterine contraction. The mechanism of action of inhaled nitrous oxide is complex, with inhibition of potassium channels in the central nervous system (CNS), the release of endogenous opioids with к-receptor activation, and anxiolysis by gamma-aminobutyric acid (GABA) receptor activation. According to the recently introduced theory, it is believed that the inhibition of N-methyl-D-aspartate (NMDA) receptor activity by nitrous oxide is mediating the primary analgesic effect [10]. Nitrous oxide elicits anxiolysis and sedation, behavioural responses similar to the effects of benzodiazepines [10]. Animal studies have shown that both nitrous oxide and benzodiazepine-mediated anxiolytic behaviours were equally sensitive to flumazenil-induced antagonism [11]. This finding indicates a possible association between the GABA receptor mechanism of benzodiazepine inhibition and the anxiolytic mechanism of nitrous oxide [11]. It remains unclear how nitrous oxide acts at the molecular level to stimulate the benzodiazepine GABA receptor binding site; however, it is hypothesised to be related to a release of benzodiazepine factors stimulating the receptor [11].

Advances in genetics revealed that subtle changes in various genes encoding for receptors could partly explain the variation in individual differences in the individual sensitivity, perception, and expression of pain, which may influence the way pain could be treated and managed [12]. Catecholamines (nor-adrenaline, adrenaline, and dopamine) are responsible for pain perception and processing, which means that they may decrease and/or increase sensitivity to pain [12]. Degradation of catecholamines is controlled by several enzymes, among which catechol-O-methyltransferase (COMT) encoded by the COMT gene in humans, together with the voltage-gated sodium channels, play a significant role in human pain perception and sensation [13,14,15]. The COMT enzyme has two major isoforms: membrane (MB-COMT) and soluble (S-COMT) bound [16,17]. According to some data, single-nucleotide polymorphism (SNP) is located in the promoter region of MB-COMT, and the second SNP is located in the promoter region of S-COMT, while three SNPs are located in the coding regions of both MB- and S-COMT. SNP rs4680 is nonsynonymous and can cause the substitution of valine (Val) with methionine (Met) at codon 158 (Val158Met), which could cause a three- to four-fold decrease in COMT activity, which may lead to enhanced dopaminergic neurotransmission with lower endogenous levels of enkephalins and thus exaggerated pain sensitivity [18,19]. Obstetricians and midwives are facing anxiety, fear, and pain during childbirth, and with a deeper genetic understanding, they are challenged by gene polymorphism interaction with pain perception and anxiety in parturients [20]. Some studies have shown that COMT polymorphism could regulate psychological and stress factors by changing the activity of catechol-O-methyltransferase (COMT) and affecting the human perception of pain [21]. COMT gene polymorphism is one genetic factor that determines different endurance and response patterns to pain or other stress [22].

The influence of the COMT polymorphisms (p.158Val/Met, c.472G/A) on inhaled nitrous oxide analgesia during labour and delivery, as well as anxiety as a personality trait, have not been evaluated according to our knowledge. The aim of this study was to investigate the association between the COMT polymorphism, anxiety trait, and pain perception in labouring women.

## 2. Materials and Methods

### 2.1. Ethical Statement

The Ethics Committee of the General Hospital Zabok and War Veterans Hospital approved this study (No. 97/2). All the women in this study signed written informed consent.

### 2.2. Study Participants

In the prospective study, 181 healthy pregnant women in the third trimester planning a vaginal birth were enrolled. The women were approached if they met the following criteria: age 18–40 years, nulliparous or parous with singleton pregnancy, vertex presentation, 37–41 weeks of gestational age. The exclusion criteria were as follows: multiple pregnancies, cardiovascular or neurological diseases, KOPD, mental or psychiatric disorders, IUGR, oligohydramnios, GDM, hypertension, pathological CTG, and breech presentation. The parturients were divided into two groups according to their personal choice regarding inhaled analgesia with nitrous oxide (*n* = 90) and without it (*n* = 91). Pregnant women were selected according to the above criteria for regular check-ups in the Department of Gynaecology in the hospital. Parturients who chose “natural” childbirth were offered childbirth without labour analgesia or inhaled analgesia with nitrous oxide as a safe and low-risk method of pain control that is clinically effective and improves the overall labour experience for parturients. Obstetricians and midwives informed parturients about the nitrous oxide anxiolytic, analgesic, and relaxing effects during labour and birth. Their enrollment in this study was based on their acceptance of nitrous oxide. The estimated number of subjects per group for testing differences in average VAS score values of 1 point (significance level 5%, test power 80%) with the expected maximum standard deviation of 2.7 points is 90. This study was conducted in accordance with the Declaration of Helsinki.

### 2.3. Data Collection

The following parameters were recorded from all parturients: age, parity, gestational age, duration of birth, weight, height, BMI, and education, as well as the new-born data: gender, birth weight (g), length (cm), Apgar scores in the 1st and 5th minutes, and umbilical vein pH. To enable maternal and foetal safety, all participants were monitored with pulse oximetry, heart rate (HR), arterial blood pressure, and foetal heart rate (FHR) monitoring, and an obstetrician was present during this study period, from the start of applying the analgesia.

### 2.4. Pain Assessment by Visual Analog Scale (VAS)

The Visual Analogue Scale (VAS) is used for pain intensity assessment. The range of the pain scale (0–100 mm) is divided into 10 points, from 0 to 10. If the score is higher, pain is more obvious. Numerical values in the pain score > 3 points represented weak pain, moderate pain was 4–6 points, and severe pain was denoted by 7–10 points. The parturients were explained how to self-evaluate pain strength using the VAS questionnaire. Pain intensity was measured in both parturient groups twice during labour using the VAS scale. The first time, at the beginning of the active phase of labour, defined as: 4–5 cm cervix dilatation and 3 contractions in 10 min lasting 1 min, and the second, referring to the pain during birth, which was evaluated after birth. In the group with nitrous oxide, the VAS score was measured before applying the analgesia.

### 2.5. Assessment of the Anxiety as a Personal Trait Using State-Trait Anxiety Inventory (STAI-T) Questionnaire

STAI—State Trait Anxiety Inventory (STAI-T) questionnaire is used for anxiety as a personality trait assessment for adults and consists of 20 claims regarding their general feelings rated from 1 point to 4 points. STAT-T questionnaire filling requirements were explained in detail, and the pregnant women filled out a form at their last check-up before labour. A higher level of pain is related to a higher score. The maximum score is 80 points. If the score range is 50–59, it indicates mild anxiety; 60–69 is moderate anxiety; and a score higher than 70 represents severe anxiety.

### 2.6. Assessment of COMT (Cathechol-O-Methytransferase) Gene Polymorphism

At the time of study enrollment, venous blood samples (4 mL) for genotyping were taken into Vacuette K3EDTA anticoagulant test tubes (Greiner Bio-One, Kremsmünster, Austria) and stored at −80 °C. The manufacturer’s protocol for DNA isolation was followed (MagMAXTM DNA Multi-Sample Ultra 2.0 Kit, Thermo Fisher, Waltham, MA, USA), performed on a King Fisher Flex analyzer, and analysed by competitive allele-specific PCR (KASP-PCR) on the ABI 7500 analyzer (Applied Biosystems, Waltham, MA, USA USA); according to the finished reaction, a signal was read by the end-point method. Specific primers for the COMT polymorphism rs4689 were commercially prepared by LGC Biotechnologies (LGC Genomics, Teddington, UK). The KASP-PCR method was used for rs4689 COMT genotyping. According to the genotype, the wild-type homozygote (Val/Val) was defined as GG, the mutant homozygote (Met/Met) was defined as AA, and the mutant heterozygote (Val/Met) was defined as GA.

### 2.7. Protocol for Inhaled Nitrous Oxide Labour Analgesia in This Investigation

Nitrous oxide (50%/50%) inhaled analgesia was administered at the request of labouring women after regular uterine contractions and when the cervix dilatation was 4–5 cm. Labouring women were educated by the obstetrician and midwives on how to use the inhaled gas mixture by themselves. Analgesia was applied by inhaling the gas mixture with normal breathing through a facial mask. Inhaling opens the demand valve for gas inflow, and with exhaling, the demand valve is closed. To achieve the successful analgesic, anxiolytic, and relaxing action of nitrous oxide, breathing has to be synchronised with labour dynamics. A labouring woman has to start inhaling 20 to 30 s prior to the beginning of contraction. Inhalation has to be stopped when the intensity of the contraction declines and started again before the new contraction starts. Nitrous oxide was not used in the expulsion phase of labour.

### 2.8. Statistical Analysis

The difference between two or more groups of parturients was tested for categorical variables using Pearson’s chi-square test or Fisher’s test if any expected frequency in the 2 × 2 (or r × c) table was less than 5. For continuous variables, the difference between two groups of parturients was tested using the Student’s *t*-test for a variable with a normal distribution (birth weight of the neonate) or the Mann-Whitney U test for variables with a distribution that deviated from the normal (other continuous variables). Differences between the three groups of parturients were tested for continuous variables using analysis of variance (ANOVA) in the case of normally distributed variables or by Kruskal-Wallis ANOVA for variables with a non-normal distribution. In the case of a significant Kruskal-Wallis ANOVA result, post hoc testing of differences between individual groups in the model was performed using the Mann-Whitney U test with Bonferroni correction for multiple comparisons. Parametric ANOVA models did not reach the appropriate level of statistical significance.

The correlation between two continuous variables was examined using the Spearman test, considering that only one variable had a normal distribution.

The potential deviation of COMT rs4680 gene polymorphisms from Hardy-Weinberg equilibrium was tested using the Hardy-Weinberg equilibrium test (https://www.stata.com/users/mcleves/genhw/genhw.hlp accessed on 22 August 2023).

Before conducting the Student’s *t*-test, the homogeneity of variances was tested by Brown and Forsythe’s modification of Levene’s test (Levene 1960; Brown and Forsythe 1974), and in ANOVA, the homogeneity of variances was tested by Bartlett’s test. The normality of the distribution of variables was tested with the Shapiro-Wilk test (Shapiro and Wilk 1965).

The association of the dependent variable (perception of pain during labour) with relevant predictors was examined in multiple linear regression models. In the model with the perception of pain during childbirth as a dependent variable, the following predictors were included: age, height and parity of the mother, sex and weight of the neonate, level of anxiety, and polymorphism of the COMT rs4680 gene.

In both models, neonate length was not included among the predictors due to a strong association with neonate weight (Spearman’s rho = 0.73; *p* > 0.001).

The level of anxiety was included in the regression model as a categorical variable. Given that anxiety was assessed as moderate in only three parturients (>2% of the total number of parturients), they were added to the group of parturients with mild anxiety for the purposes of the regression analysis.

## 3. Results

### 3.1. The Influence of Pain Relief with N_2_O during Delivery on the Perception of Pain during Delivery

Table 1 shows the characteristics of the examined group of parturient women, both for all parturients as well as for the subgroups with regard to analgesia with N_2_O during childbirth. Statistically significant differences between parturients with and without analgesia were found in the following categories: age, parity, and perception of pain after childbirth. The group of parturients who received analgesia were, on average, three years younger, with lower parity (in this group, there was a 1.7 times higher frequency of primiparous women, as well as a three times lower frequency of multiparous women who had their third, fourth, or fifth birth), and with a lower perception of pain at the end of childbirth compared to the parturients who did not get analgesia.

The two groups of parturients did not differ with regard to their height, the duration of gestation, the sex, weight, and length of the neonate, the level of anxiety, or the frequency of tested COMT gene polymorphisms.

#### 3.1.1. Pain Relief with N_2_O and the Perception of Pain at the Beginning of Delivery

The perception of pain at the beginning of delivery was connected only with the perception of pain at the end of delivery and with the sex of the neonate. A positive correlation between the perception of pain at the beginning of delivery and the perception of pain at the end of delivery was observed both in the parturients who received no analgesia during childbirth (*p* = 0.001) and in the parturients who received analgesia (rho = 0.380, *p* > 0.001). Among parturients with analgesia, the perception of pain at the beginning of delivery was slightly higher in mothers who gave birth to male children (median 4, interquartile range: 3–5) compared to parturients who gave birth to female children (median 3, interquartile range: 2–4) (*p* = 0.029). Among those giving birth without analgesia, this difference was not observed.

#### 3.1.2. Pain Relief with N_2_O and the Perception of Pain at the End of Delivery

Although pain relief did not have any effect on the perception of pain at the beginning of delivery, almost all the parturients who did not receive analgesia reported very strong pain (98%) at the end of delivery, while among those with pain relief, 22% reported only moderate pain, and the rest reported very strong pain (78%). Among the parturients who did not receive pain relief, the perception of pain at the end of delivery was positively correlated with the length of the neonate (*p* = 0.013). Parturients with analgesia, this association was not observed. Other predictors were not statistically significantly related to the perception of pain at the end of delivery.

The influence of pain relief on the perception of pain at the end of delivery was also examined in a linear regression analysis model. Lower pain perception at the end of delivery remained significantly associated with analgesia, with control of potential influencing factors (age, height, and parity of the mother; sex and weight of the baby; the level of anxiety; COMT polymorphism). Pain relief was associated with a 0.92-unit lower pain perception compared to the parturients without analgesia (95% confidence interval from −1.27 to −0.57). Other predictors included in the analysis did not have a significant association with the perception of pain at the end of delivery, with the exception of the GA polymorphism of the COMT gene.

### 3.2. The Perception of Pain during Delivery with Regard to COMT Polymorphism

Among the 181 pregnant women, there were 40 women (22%) in the GG group, 95 (53%) in the GA group, and 46 (25%) in the AA group. The allele distribution of COMT was found to be in line with the Hardy-Weinberg equilibrium.

#### The Perception of Pain during Delivery with Regard to COMT Gene Polymorphism

Univariate analysis did not show significant differences in the perception of pain during delivery and in the duration of delivery between the parturients with different polymorphisms of the COMT gene, both among all the mothers and in the subgroups of the mothers with regard to N_2_O pain relief.

In the multiple linear regression model, with control for pain relief and other potential influencing factors (age, height, and parity of the mother, sex and weight of the baby, the duration of labour, the level of anxiety), however, a negative association of the perception of pain with the GA polymorphism of the COMT gene compared to the wild type (GG polymorphism) was observed. The GA polymorphism of the COMT gene was associated with a 0.46-unit lower perception of pain compared to the parturients with the wild type (GG).

With the exception of analgesia, the other predictors were not significantly associated with the perception of pain at the end of delivery. Moreover, no statistically significant interactions were found between the effect of pain relief and COMT gene polymorphism on the perception of pain at the end of delivery.

### 3.3. The Relationship between Anxiety and the Perception of Pain during Delivery

#### 3.3.1. Anxiety and the Perception of Pain during Delivery

With regard to the perception of pain during delivery, univariate analysis showed a statistically significant difference between the parturient women without anxiety and those with mild anxiety only for the perception of pain at the end of delivery in the parturients who did not receive N_2_O pain relief (Table 2). The medians of these two groups of women, however, did not differ, and the association between anxiety and the perception of pain at the end of delivery was not observed in the multiple regression analysis model controlled for other predictors (age, height and parity of the mother, sex and weight of the baby, analgesia, polymorphism of COMT) (Table 3). Moreover, no statistically significant interaction was found between analgesia and the level of anxiety in relation to the perception of pain at the end of delivery.

#### 3.3.2. Association of COMT Gene Polymorphism with Anxiety

The level of anxiety differed considering the polymorphism of the COMT gene. Among all the parturients, a discrete but statistically significant difference was found in the Kruskal-Wallis ANOVA for the COMT gene polymorphism; however, mutual comparisons between the groups (GG vs. GA; GG vs. AA; GA vs. AA) did not reach statistical significance.

In the parturients with pain relief, however, it was observed that those with the AA polymorphism (mutant homozygote) had 1.5 points less on the STAI-T (State-Trait Anxiety Inventory) anxiety scale compared to the GG polymorphism (wild type) and 2.5 points less compared to the GA polymorphism (mutant heterozygote). The difference reached statistical significance for the comparison of AA with GA polymorphism (Table 4).

## 4. Discussion

We investigated the association of the COMT polymorphism with anxiety as a personality trait and pain perception in parturients with inhaled nitrous oxide.

The analgesic effect of nitrous oxide is difficult to quantify properly and uniformly as pain relief. There are also missing well-defined measures of the analgesic effectiveness of nitrous oxide, according to the systematic review by Rosen [23] and Likis [24]. The authors were unable to draw conclusions on the degree of pain relief afforded by nitrous oxide. There were a few trials where nitrous oxide efficacy measured by VAS was compared with labouring pain with no analgesia performed. Most studies have focused on the comparison of different types of analgesia and pain perception among them, e.g., nitrous oxide vs. epidural.

We observed the effect of analgesia in the group of women during delivery who received analgesia with nitrous oxide at the end of the active phase of delivery. In the parturients with analgesia, 22% rated the pain at the end of delivery as medium (VAS score 4–7), while the other 78% rated the pain as very strong (VAS 8–10). In contrast, 98% of the parturients who were not to get analgesia with nitrous oxide rated the pain at the end of delivery as very strong.

Among those who did not receive analgesia, we observed that the perception of pain at the end of the active phase of delivery was positively correlated with the length of the neonate (*p* = 0.013). The correlation between the baby’s length and stronger pain at the end of delivery, found in this study, can be explained by the fact that taller newborns likely have a greater head circumference, which in turn adds to increased pain. Unfortunately, head circumference was not measured in this study; therefore, we can only hypothesise that a larger head circumference may contribute to stronger pain at the end of delivery. While among those who received nitrous oxide, this connection was not observed, we can therefore conclude that the effect of analgesia was so effective that the length of the neonate in the group with inhaled analgesia did not have a statistically significant effect on pain during delivery.

To obtain the best possible analgesic effect from the use of inhaled nitrous oxide during labour, appropriate patient education and follow-up are needed, as found by Talebi et al., who noted that appropriate nitrous oxide application combined with precise patient instructions and coaching during labour reduced the VAS score by 20 mm (2 points) compared to the control group [25].

In our study, the analgesic effect of nitrous oxide was associated with a 0.92 unit (9.2 mm) lower pain perception compared to those who did not receive analgesia, which was a worse result than obtained by Talebi et al. [26], The difference in our study results and in Talebi et al. could be explained by the COVID-19 pandemic and the fewer face-to-face contacts of pregnant women with healthcare providers who had to educate the patients online or by phone calls.

In our study, nitrous oxide was applied intermittently at a concentration of 50%, and the results were similar to those of Westling et al. They examined different concentrations of nitrous oxide, 40% and 70%, and 100% oxygen intermittently and 40% nitrous oxide continuously. In that study, it was shown that the analgesic effect depends on the dose; 40% shows a decrease in VAS score by 10 mm with intermittent use, with continuous use by 18 mm, and with continuous application of 40% nitrous oxide, the VAS score was lower by 27 mm [26]. Intermittent application of nitrous oxide requires increased concentration and attention from the parturient regarding the recognition of the upcoming contraction in order to be able to apply the nitrous oxide and expect the next contraction before the onset of pain, which occurs with a time delay of 15 s, which is difficult for some parturients. Many comments were recorded in a study by Richardson et al. where the women on the first day post-delivery who delivered with nitrous oxide analgesia said that analgesia was incomplete but that patient expectations were met [27]. Many women stated that the technique enhanced their coping with labour by shifting attention and reducing anxiety. A significant minority said that it was consistent with their birth plan (some parturients noted that nitrous oxide enhanced their “natural childbirth”) [27]. Women who decided for vaginal, “natural” birth could get an effective alternative to neuraxial analgesia or a caesarean section with nitrous oxide [28]. Nitrous oxide offers a pain relief method that is very safe for the mother, foetus, and neonate.

Labour analgesia is used for the alleviation of labour pain; however, the analgesic effect varies among parturients. Our study showed that the COMT gene polymorphism GA was correlated with the level of anxiety and pain among parturients. In the multiple linear regression model in this study, a negative association of pain perception with GA (mutant heterozygotes) polymorphism of the COMT gene compared to the wild-type (GG polymorphism) was observed. The GA polymorphism of the COMT gene was associated with a 0.46-unit lower pain perception compared to the group of parturients with the wild type (GG) of the COMT gene, while the interaction between the analgesic effect of nitrous oxide and the COMT gene polymorphism was not observed.

Studies have shown that genetic factors are closely related to labour anxiety and pain perception. One study has revealed that the activity of COMT is an important regulator of pain sensitivity [29]. Another study has also indicated that the Val158Met mutation of the COMT gene can affect the sensitivity of the body to pain by altering the activity of COMT and thus changing the hormone levels and the psychological state [30]. Ren et al. showed in their study results that the pain score in groups AA and GA was significantly higher than in the GG group. According to Tang et al., the pain score of women with the AA genotype was higher than that of GG and GA [31]. The findings of our study exhibit discrepancies, and it is plausible that in the studies conducted by Ren and Tang, women received analgesia with fentanyl and dexmedetomidine, in contrast to our study, where nitrous oxide was administered and a limited number of participants were involved.

Psychological factors associated with labour pain experience are, for example, coping strategies and expectations [32]. Numerous studies have evaluated psychological factors associated with labour pain, but not many studies have evaluated anxiety as a personality trait as a possible predictor of labour pain perception.

In the Dabo Pettersson et al. study, women with an increased self-rated pain score according to the Spilberger Stait-Trait Anxiety Inventory (STAI-T) reported higher self-rated pain prior to labour analgesia compared to women with a low STAI-T [6].

This study showed a statistically significant difference (*p* = 0.029) between non-anxious and mildly anxious parturients in the perception of pain at the end of the active phase of delivery, but only in the parturients who received analgesia with nitrous oxide. Due to the positive analgesic effect of nitrous oxide, the degree of anxiety did not affect the perception of pain among the parturients with analgesia. The obtained results are in accordance with the research of Vallejo et al., in which no significant difference was observed in the analgesic effect of nitrous oxide in parturients with low anxiety [33]. In our study, no statistically significant correlation was found between the degree of anxiety and the perception of pain at the beginning of delivery. However, it should be noted that in this study, we had healthy pregnant women without anxiety or with only mild anxiety. The obtained data are in accordance with the data from the literature that suggest a direct connection between the degree of prenatal anxiety and maximum pain during delivery [34]. In addition, pain scorings were collected retrospectively, i.e., within 24 h of delivery, and recall bias may have influenced the pain severity ratings.

The level of anxiety in parturients differed with regard to the polymorphism of the COMT gene, but only in parturients with analgesia. In these pregnant women, it was observed that those with the AA polymorphism had 1.5 points less on the STAI-T anxiety scale compared to the GG polymorphism and 2.5 points less compared to the GA polymorphism (mutant heterozygote). The difference reached statistical significance for the comparison of AA with GA polymorphism.

In contrast to this study, Tang et al. have shown that polymorphism in the GA COMT gene affect the degree of anxiety and pain before the administration of analgesia during delivery. In the same study, the author compared the effectiveness of dexmedetomide on the three genotypes, and the results showed that there was no significant difference in the degree of anxiety and pain between the GG, GA, and AA polymorphisms, while the GA polymorphism of the COMT gene did not affect the analgesic effect of dexmedetomide in delivery, which is similar to the results we obtained in our study in which we applied nitrous oxide. Xiaohong et al. found in their study that prenatal anxiety was significantly higher in the AA group of women in contrast to those in the GG group [35].

In this study, we investigated the association of the COMT gene polymorphism with anxiety as a personality type and pain perception in parturients who received pain relief with nitrous oxide and deepened our knowledge about anxiety as a personality state as well as analgesia with nitrous oxide during vaginal births.

This study has limitations due to the small number of participants and the analysis of only one mutation, SNP rs 4860. There was no interference with other SNPs of the COMT gene and other genes that influence pain and analgesic efficacy during delivery, which could explain our study results. An increasing number of studies strongly suggest that genetic predisposition plays an important role in pain and pain-related mechanisms [36]. Gender-genotype and ethnicity-dependent genetic interactions have been shown to exist, indicating that we cannot generalise findings from different cohorts and extrapolate from one study population to another [37]. Other limitations that could impact results are self-rated pain and VAS scores collected within 24 h after delivery. VAS score results are subjective, affected by different expectations, and driven by strong emotions. Moreover, cultural, physiological, and social components play important roles in pain perception during childbirth, which were not taken into consideration in this study.

## 5. Conclusions

The COMT gene polymorphism was associated with pain perception and anxiety among parturients. In women with mild anxiety, nitrous oxide has been found to be effective in reducing anxiety during labor. Additionally, it may provide mild pain relief for some parturient. Since the exact effect of genes on analgesia during delivery is unknown, additional multicentric research is needed with a large number of parturients, including gene polymorphisms and their co-occurrences. This would allow us in the future to demonstrate, based on genetic research, which analgesic drug would be most effective for a particular group of parturients. This could allow them to overcome anxiety, fear, and discomfort as efficiently as possible, or make them acceptable, especially for those who desire a vaginal (“natural”) birth, and enable a more precise personal approach.

## Figures and Tables

**Table 1:** The characteristics of the examined group of parturient women with regard to pain relief during delivery.

	All Mothers	Without Pain Relief	With Pain Relief	Differences between Groups	
				χ^2^/*t*-Test/ z Value	*p*
Number of mothers	181	90	91		
Age [years]	30 (26–34) (21–44)	31 (27–36) (22–44)	28 (26–32) (21–42)	2.83 ^d^	0.005
Mother’s height [cm]	165 (162–170) (153–183)	165 (163–169) (156–183)	165 (162–170) (153–182)	0.458 ^d^	0.648
Mother’s parity					
first childbirth	93 (51)	34 (38)	59 (65)	15.8 ^a^	<0.001
second childbirth	59 (33)	34 (38)	35 (27)		
third+ childbirth	29 (16)	22 (24)	7 (8)		
Weeks of gestation					
38 weeks	22 (12)	11 (12)	11 (12)	5.16 ^b^	0.277
39 weeks	52 (29)	32 (36)	20 (22)		
40 weeks	36 (20)	17 (19)	19 (21)		
41 weeks	64 (35)	28 (31)	36 (40)		
42 weeks	7 (4)	2 (2)	5 (5)		
Male sex of the baby	91 (50)	42 (47)	49 (54)	0.933 ^a^	0.334
Baby’s weight [g]	3530 ± 445 (2200–4750)	3547 ± 481 (2200–4650)	3514 ± 408 (2345–4750)	0.500 ^c^	0.618
Baby’s length [cm]	51 (50–51) (45–56)	51 (50–52) (45–56)	50 (50–51) (45–53)	0.746 ^d^	0.457
Perception of pain at the beginning of delivery	3 (3–5) (1–7)	3 (3–5) [1–7)	4 (2–5) (2–7)	1.58 ^d^	0.115
low pain	94 (52)	53 (59)	41 (45)	3.47 ^a^	0.063
medium pain	87 (48)	37 (41)	50 (55)		
very strong pain	0	0	0		
Perception of pain at the end of delivery	9 (8–10) (4–10)	9 (9–10) [6–10)	8 (8–9) (4–10)	5.22 ^d^	<0.001
low pain	0	0	0		
medium pain	22 (12)	2 (2)	20 (22)	16.5 ^a^	<0.001
very strong pain	159 (88)	88 (98)	71 (78)		
Level of anxiety	46 (44–49) (33–67)	46 (44–49) (38–60)	46 (44–49) (33–67)	0.013 ^d^	0.990
no anxiety	142 (78)	72 (80)	70 (77)	0.467 ^b^	0.836
mild anxiety	36 (20)	17 (19)	19 (21)		
moderate anxiety	3 (2)	1 (1)	2 (2)		
COMT gene					
GG (wild type)	40 (22)	19 (21)	21 (23)	1.14 ^a^	0.565
GA (mutant heterozygote)	95 (53)	45 (50)	50 (55)		
AA (mutant homozygote)	46 (25)	26 (29)	20 (22)		

The results are presented as frequencies (percentage) for categorical variables; as arithmetic mean ± standard deviation (range) for a normally distributed variable; or as the median (interquartile range) for variables with a distribution that deviates from normal. The difference between the two groups of women was tested using ^a^ Pearson’s chi-test; ^b^ Fisher test; ^c^ Student’s test (for variables with a normal distribution), or ^d^ Mann-Whitney U test (for variables with a distribution that deviates from normal). The results have been considered statistically significant if *p* < 0.05.

**Table 2:** The perception of pain during delivery and the duration of delivery with regard to the level of anxiety and pain relief during delivery.

	Level of Anxiety		Differences between Groups	
	No Anxiety	Mild Anxiety	Pearson’s or K-W χ^2^	*p*
All Mothers				
Number of mothers	142	39		
Perception of pain at the beginning of delivery	4 (3–5) (1–7)	4 (3–5) (2–6)	−0.50 ^c^	0.611
mild pain	75 (53)	19 (49)	0.21 ^a^	0.650
medium pain	67 (47)	20 (51)		
very strong pain	0	0		
Perception of pain at the end of delivery	9 (8–10) (4–10)	9 (8–10) (5–10)	1.25 ^c^	0.215
mild pain	0	0		
medium pain	16 (11)	6 (15)	0.49 ^b^	0.326
very strong pain	126 (89)	33 (85)		
Mothers without pain relief				
Number of mothers	72	18		
Perception of pain at the beginning of delivery	3 (3–5) (1–7)	3 (3–5) (2–6)	−0.09 ^c^	0.939
mild pain	43 (60)	10 (56)	0.10 ^a^	0.748
medium pain	29 (40)	8 (44)		
very strong pain	0	0		
Perception of pain at the end of delivery	9 (9–10) (6–10)	9 (8–10) (7–10)	2.21 ^c^	0.029
mild pain	0	0		
medium pain	1 (1)	1 (6)	1.15 ^b^	0.362
very strong pain	71 (99)	17 (94)		
Mothers without pain relief				
Number of mothers	70	21		
Perception of pain at the beginning of delivery	4 (2–5) (2–7)	4 (3–4) (2–6)	−0.37 ^c^	0.721
mild pain	32 (46)	9 (43)	0.05 ^a^	0.817
medium pain	38 (54)	12 (57)		
very strong pain	0	0		
Perception of pain at the end of delivery	8 (8–9) (4–10)	8 (8–9) (4–10)	−0.26 ^c^	0.800
mild pain	0	0		
medium pain	15 (21)	5 (24)	0.05 ^b^	0.515
very strong pain	55 (79)	16 (76)		

The difference between the two groups of women was tested using ^a^ Pearson’s chi-square test; ^b^ Fisher test; ^c^ Student’s test, or a Mann-Whitney U test. The results have been considered statistically significant if *p* < 0.05.

**Table 3:** Multiple linear regression analysis of pain perception at the end of delivery.

Predictors:	Regression Coefficient	95% Confidence Interval	*p*
Pain relief	−0.918	−1.27; −0.568	<0.001
Mother’s age	−0.008	−0.045; 0.029	0.662
Mother’s parity			
first childbirth	reference category		
second childbirth	−0.011	−0.424; 0.401	0.957
third+ childbirth	0.258	−0.312; 0.828	0.373
Mother’s height	−0.007	−0.037; 0.023	0.659
Baby’s weight	6.5 × 10^−5^	−3.4 × 10^−4^; 4.7 × 10^−4^	0.755
Male sex of the baby (vs. female)	−0.127	−0.474; 0.220	0.471
Mild anxiety (vs. no anxiety) *	−0.146	−0.555; 0.263	0.481
COMT gene			
GG (wild type)	reference category		
GA (mutant heterozygote)	−0.460	−0.885; −0.034	0.034
AA (mutant homozygote)	−0.296	−0.788; 0.196	0.236

The results have been considered statistically significant if *p* < 0.05. *: Only three out of the total number of parturients (<2%) had moderate anxiety, so they were grouped with those experiencing mild anxiety for the regression analysis.

**Table 4:** The level of anxiety with regard to COMT rs4680 gene polymorphism and pain relief during delivery.

	COMT rs4680 Gene Polymorphism			Differences between Groups	
	GG	GA	AA	Pearson’s or K-W χ^2^	*p*
All mothers					
Number of mothers	40	95	46		
Level of anxiety	46 (43.5–48.5) (40–67)	47 (45–50) (33–63)	45 (43–49) (39–56)	6.36 ^c^	0.042
no anxiety	33 (83)	70 (74)	39 (85)	2.76 ^a^	0.252
mild anxiety	7 (18)	25 (26)	7 (15)		
Mothers without pain relief					
Number of mothers	19	45	26		
Level of anxiety	46 (42–49) (40–53)	46 (45–49) (38–60)	47 (43–49) (39–56)	0.52 ^c^	0.772
no anxiety	16 (84)	34 (76)	22 (85)	1.11 ^b^	0.651
mild anxiety	3 (16)	11 (24)	4 (15)		
Mothers without pain relief					
Number of mothers	21	50	20		
Level of anxiety	46 ^ab^ (44–48) (40–67)	47 ^a^ (46–50) (33–63)	44.5 ^b^ (43–46) (40–53)	8.58 ^c^	0.014
no anxiety	17 (81)	36 (72)	17 (85)	1.61 ^b^	0.531
mild anxiety	4 (19)	14 (28)	3 (15)		

The difference between the three groups of women was tested using ^a^ Pearson’s chi-square test; ^b^ Fisher test, or ^c^ Kruskal-Wallis ANOVA test. The results have been considered statistically significant if *p* < 0.05. The letters in superscript indicate statistically significant differences between the groups, with a Bonferroni correction for multiple comparisons (0.017).

## Data Availability

The datasets generated and analysed in the current study are available from the corresponding author on reasonable request.
